# Simulation of Atmospheric Dispersion of Elevated Releases from Point Sources in Mississippi Gulf Coast with Different Meteorological Data

**DOI:** 10.3390/ijerph6031055

**Published:** 2009-03-11

**Authors:** Anjaneyulu Yerramilli, Challa Venkata Srinivas, Hari Prasad Dasari, Francis Tuluri, Loren D. White, Julius M. Baham, John H. Young, Robert Hughes, Chuck Patrick, Mark G. Hardy, Shelton J. Swanier

**Affiliations:** 1 Trent Lott Geospatial Visualization Research Centre, Mississippi e-Center, Jackson State University, 1230 Raymond Road, Jackson MS 39204, USA; E-Mails: cvsri@igcar.gov.in (C.V.S.); hari.dasari@jsums.edu (H.P.D.); francis.tuluri@jsums.edu (F.T.); julius.m.baham@jsums.edu (J.M.B.); john.h.young@jsums.edu (J.H.Y.); robert.l.huges@jsums.edu (R.H.); cpatrick@jsums.edu (C.P.); 2 Department of Physics, Atmospheric Science and General Sciences, Jackson State University, 1400, Lynch Street, Jackson MS 39217, USA; E-Mail: white@twister.jsums.edu; 3 College of Science, Engineering &Technology, Jackson State University, 1400 Lynch Street, Jackson MS 39217, USA; E-Mails: mark.g.hardy@jsums.edu (M.G.H.); sswanier@jsums.edu (S.J.S.)

**Keywords:** Meteorological data, HYSPLIT model, dispersion estimates

## Abstract

Atmospheric dispersion calculations are made using the HYSPLIT Particle Dispersion Model for studying the transport and dispersion of air-borne releases from point elevated sources in the Mississippi Gulf coastal region. Simulations are performed separately with three meteorological data sets having different spatial and temporal resolution for a typical summer period in 1–3 June 2006 representing a weak synoptic condition. The first two data are the NCEP global and regional analyses (FNL, EDAS) while the third is a meso-scale simulation generated using the Weather Research and Forecasting model with nested domains at a fine resolution of 4 km. The meso-scale model results show significant temporal and spatial variations in the meteorological fields as a result of the combined influences of the land-sea breeze circulation, the large scale flow field and diurnal alteration in the mixing depth across the coast. The model predicted SO_2_ concentrations showed that the trajectory and the concentration distribution varied in the three cases of input data. While calculations with FNL data show an overall higher correlation, there is a significant positive bias during daytime and negative bias during night time. Calculations with EDAS fields are significantly below the observations during both daytime and night time though plume behavior follows the coastal circulation. The diurnal plume behavior and its distribution are better simulated using the mesoscale WRF meteorological fields in the coastal environment suggesting its suitability for pollution dispersion impact assessment in the local scale. Results of different cases of simulation, comparison with observations, correlation and bias in each case are presented.

## 1. Introduction

Increasing urbanization, industrial growth and population expansion in coastal areas necessitates accurate air pollution dispersion estimates. A number of regional-scale meteorological conditions and prevailing winds influence the pollutant trajectories and ground level concentrations. Coastal regions are particularly interesting as topographic variations and land-sea interface govern the local flow. Pollutant plumes in the coastal zones are influenced by development of meso-scale sea breeze circulations as a result of differential heating of the land and water surfaces [1,2]. Differential land-sea temperatures and the incidence of local circulations initiate development of internal boundary layer (IBL), which has a critical effect on dispersion [3,4]. These local effects need to be accounted in the coastal dispersion simulation for realistic estimations of pollutant concentrations. Proper meteorological inputs are needed to obtain realistic estimation of concentrations. At present several sources of meteorological data are available at different spatial and temporal resolution to study long-range transport or the local scale dispersion. Numerous studies show the spatial and temporal resolution of meteorological data is an important factor in accurate estimation of plume trajectories and concentration [5,6]. Nasstrom and Pace reported in 1998 that higher resolution meteorological data lead to improvement in meso-scale dispersion through better representation of flow features [7]. Draxler, in 2006, reported application of meteorological fields produced by Penn State University (PSU)/National Center for Atmospheric Research (NCAR) mesoscale meteorological model (MM5) improved dispersion calculations on urban scales over the forecast fields from global models [8]. Several studies reported application of numerical meso-scale models for driving the dispersion models in complex terrain to capture the complex flow and meteorological parameters essential in dispersion estimations [9–11]. Segal *et al*. in 1998, applied a coupled mesocale atmospheric dispersion model to study the ground level SO_2_ concentrations from major elevated sources in Southern Florida [12]. Their study revealed that the local sea-breeze circulations lead to complex meso-scale dispersion pattern causing higher concentrations on the east coast. Usually meteorological fields (analyses/forecasts) commonly available from operational weather agencies are employed in dispersion estimations due to their availability in near real-time. Secondly, meso-scale models are computationally expensive and need to be designed carefully with appropriate input data such as terrain/topographic information, physics schemes and initial/boundary condition data for a selected domain which preclude their application for dispersion estimations when data is generally available at a desired scale from operational weather agencies. However it is necessary to evaluate such commonly available meteorological data for application in a specific location especially in challenging coastal environments.

**Figure 1.**
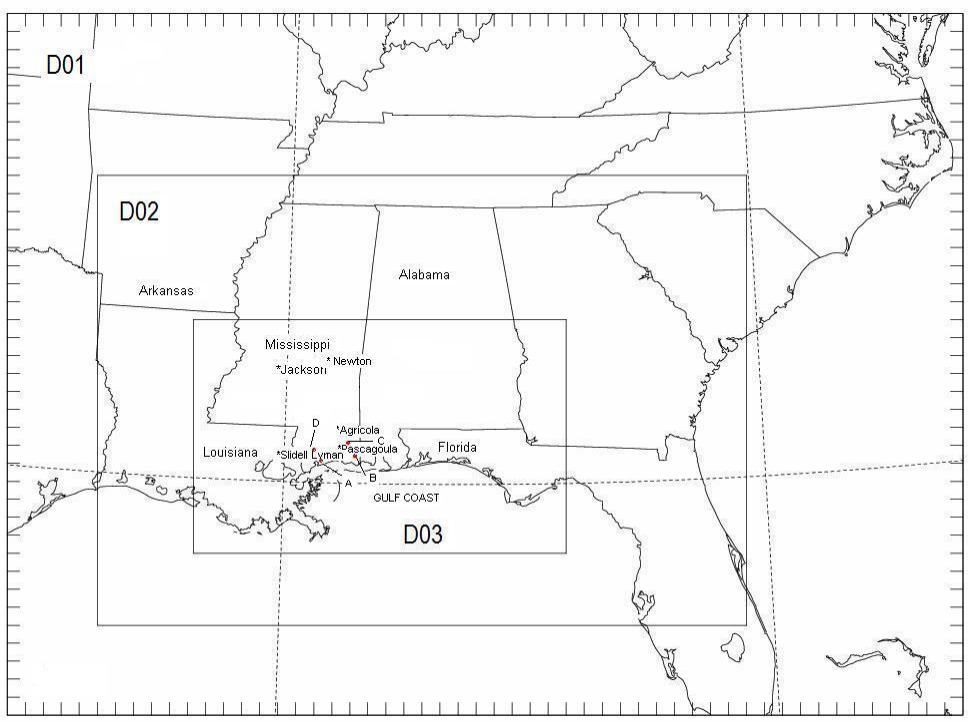
Location of the study area Mississippi Gulf coast and the domains used in the WRF model simulation.

Southern Mississippi (MS) has densely populated urban regions at Biloxi, Gulfport, Harrison located along the Gulf Coast. Several industries situated along the MS coast emit elevated releases. Considerable meso-scale forcing exists in the Mississippi Gulf coast due to differential temperatures across the coast, variability in the land use and complex topography. This area, located to the east of Louisiana complex coastline (Figure 1), experiences typical coastal atmospheric conditions in terms of flow field variability, temperature and mixing depth characteristics. In this work, a numerical modeling approach has been adopted to examine the environmental SO_2_ concentrations from some of the significant elevated sources in the MS Gulf coast region. A few commonly available meteorological model data and a meso-scale model prognosis that vary in their temporal and spatial resolution are tested using the Hybrid Single Particle Lagrangian Integrated trajectory model (HYSPLIT) [13] to simulate pollutant releases in the coastal environment. The analysis data sets included the NCEP Final Analysis (FNL), and the NCEP Eta Data Assimilation System (EDAS) analysis [14,15,16]. A simulation was conducted using the Weather Research and Forecast (WRF) meso-scale model to study the aspects of the coastal atmospheric circulations in the Mississippi Gulf coast [17]. The meteorological outputs from that simulation study are used to provide the high resolution wind and turbulence fields as the third data set in the present study. Summer synoptic conditions are considered to model the concentrations as the meteorological patterns during summer season are associated with a high pressure in the Gulf of Mexico, a weak synoptic forcing and significant land-sea temperature contrast conducive to the development of local meso-scale circulations in Mississippi. The objective of the work is to study the complexity of the elevated plume dispersion in distance ranges of a hundred kilometers under the combined influences of the local land-sea breeze circulation and synoptic flow.

Since the interest here is to find the differences that arise in the concentration patterns in the three different cases of input data, simulations are compared to ground-level air concentrations to assess the relative performance of each of the meteorological data sets. The description of different meteorological data sets used for the study is given in Section 2, the dispersion model is described in Section 3 and the intercomparison of dispersion calculations is given in Section 4.

## 2. Brief Description of Numerical Models

### 2.1. Meteorological Models and Data Generation

The modeling period was selected as June 1–3, 2006 during a summer synoptic condition. A moderate easterly synoptic flow associated with a high pressure over the subtropical North Atlantic Ocean prevailed during this period. The coarse meteorological data is chosen from two sources i.e., the NCEP FNL analyses, and the EDAS Eta analyses. The FNL analyses is prepared by combining observations with short-range forecasts from a global model and the data are available on the surface and 26 vertical levels from 1,000 mb to 10 mb at a spatial resolution of roughly 100 km at 6 h intervals. EDAS is a regional analyses for North America based on the Eta regional model. These data are available at 3 h intervals on Eta 212 grid at a spatial resolution of 40 km on 26 vertical levels from 1,000 mb to 50 mb. To obtain high-resolution meteorological fields a simulation is conducted with the Advanced Research WRF (ARW) meso-scale model (V2.2) [18]. The model consists of fully compressible non-hydrostatic equations and the prognostic variables include the three-dimensional wind, perturbation quantities of potential temperature, geo-potential, surface pressure, turbulent kinetic energy and scalars (water vapour mixing ratio, cloud water). The model vertical coordinate is terrain-following hydrostatic pressure and the horizontal grid is Arakawa C-grid staggering. A 3^rd^ order Runge-Kutta time integration is used in the model.

### 2.2. WRF Model Domains and Initialization

The details of the meso-scale simulation are given in [17] and are briefly described here. The model is designed with three nested grids (36, 12 and 4 km) and with 34 vertical levels. The outer domain covered the South-central US and the surrounding Atlantic Ocean (Figure 1). The inner finer grid covered the Mississippi Gulf Coast off Louisiana above the Gulf of Mexico. The 36 km grid (54 by 40 points) is centered at 30.8 N, 85.3 E and the finest 4 km grid (187 by 118 points) is centered at 30.96 N, 87.5 E and covers the Mississippi Gulf Coast (Figure 1). The second and third nests are two way interactive. The model physics options used are Kain-Fritsch scheme [19] for convective parameterization, WRF Single Moment Class 3 (WSM3) simple ice scheme for explicit moisture [20], Yonsei University non-local scheme for boundary layer [21], standard five-layer soil model [22], Dudhia scheme for short wave radiation [23] and the Rapid Radiative Transfer Model [24] for long-wave radiation processes. The model is initialized at 00 UTC 1 June and integrated for 48 hours using FNL data for initial and boundary conditions. Four dimensional data assimilation (FDDA) [25,26,27] grid nudging is performed for the first 12 h period on all grids for temperature, mixing ratio and wind on the model grids using the NCEP ADP (Atmospheric Data Project) surface and upper air observations. The WRF model is run in the data assimilative mode for the first 12 h period (after enhancing the initial / boundary conditions with incorporation of surface / upper air observations), and then in pure forecast mode thereafter. The nudging coefficients used are 2.5×10^−5^ for temperature and wind and 1.0×10^−5^ for mixing ratio. The USGS topography and vegetation data (25 categories) and FAO Soils data (17 categories) with resolutions 5m, 2m and 30 sec (0.925 km) were used to define the lower boundary conditions. Various options used in WRF model are given in Table 1.

### 2.3. Dispersion Model

The Hybrid Single-Particle Lagrangian Integrated Trajectory (HYSPLIT) model [13] developed by the Air Resources Laboratory is used to simulate the dispersion of airborne pollutant releases. It computes both simple trajectories and complex dispersion and deposition simulations using puff and or particle approaches. The dispersion computation consists of three components: particle transport by the mean wind, a turbulent transport component, and the computation of air concentration. Pollutant particles are released at the source location and passively follow the wind field. The mean particle trajectory is the integration of the particle position vector in space and time. In particle mode, the turbulent component of the motion defines the dispersion of the pollutant cloud and it is computed by adding a random component to the mean advection velocity in each of the three-dimensional wind component directions. The vertical turbulence is computed from the wind and temperature profiles and the horizontal turbulence is computed from the velocity deformation. The meteorological fields needed in the model are u,v,w (horizontal, vertical wind components), T(temperature), Z (height) or P (pressure), surface pressure (Po) and the optional fields moisture and vertical motion. These gridded three dimensional fields are linearly interpolated in space and time to the particle’s position.

The advection of a particle or puff is computed from the grid scale three-dimensional velocity vectors obtained from the meso-scale model. The horizontal turbulent velocity components at any given time are computed from the turbulent velocity components at the previous time, an autocorrelation coefficient that depends upon the time step, the Lagrangian time scale, and a computer generated random component. The lagrangian time scales T_Lw_ (vertical) = 100 s and T_Lu_ (horizontal) = 10,800 s are assumed to be constant. These values result in a random walk vertical dispersion for most of the longer time steps. Turbulent mixing is calculated using a diffusivity approach based upon vertical stability estimates and the horizontal wind field deformation. The stability estimates are based on surface fluxes when available or the wind and temperature profiles otherwise. Pollutant concentrations are estimated as the integrated mass of individual particles as they pass through the concentration grid which is a matrix of cells, each with a volume defined by its dimensions.

### 2.4. Dispersion Simulation

Three simulations were conducted with the HYSPLIT model using i) FNL analysis ii) Eta analysis and iii) WRF model outputs. Results are compared with observations to study the impact of the spatial and temporal resolution of the mean wind on the dispersion pattern. Dispersion simulation is done over a range of 100 km around the sources. A horizontal grid of 2° × 2° with resolution of 0.01° × 0.01° (approximately 1 km × 1 km) and with eight vertical levels 25, 50, 100, 200, 500, 1,000, 2,000 and 5,000 m above ground level (AGL) is considered in HYSPLIT. The emissions data is taken from the Mississippi Department of Environmental Quality (MDEQ) and comprises normal annual average for the respective sources. From a cluster of elevated point sources located along the MS coast, four major sources are considered in the present study. These are Mississippi Power Company-Plant Jack Watson (MPJ), Chevron Products Company- Pascagoula Oil Refinery (CR), Mississippi Power Company- Plant Victor Daniels (MPV) and Dupont Delisle Facility (DDF) (Table 2).

The dispersion calculations are made for SO_2_ species and no seasonal or diurnal variations in the emissions are considered in the present study. Also the plume rise due to plume effluent velocity and plume temperature is not considered in the present study. The point sources considered have exit velocities since power plant plumes are certainly buoyant. The buoyant plumes rise to higher heights before being subjected to downwind transport and dispersion. The plume rise for these buoyant plumes is expected to impact the trajectory paths and concentration results since there is considerable vertical variation in winds and temperature with height. A detailed calculation of plume rise could be done in future work using the next version of HYSPLIT which incorporates the complete plume rise equations. The pollutant plume is treated as top-hat puffs in the horizontal and particle in the vertical. A total of 500 particles or puffs are released during one release cycle with a maximum of 10,000 particles permitted to be carried at any time during the simulation (Table 3). The vertical turbulence mixing is computed using a diffusivity approach based upon vertical stability estimates. Although there are several methods for the calculation of horizontal diffusivity such as isotropic similarity based on turbulent fluxes or temperature/ wind profiles (i.e., gradient Richardson number), in the present study the standard velocity deformation method is used as it is relatively simple and requires only the wind field which is commonly available in all the three data sets used in the study. Ground level concentrations are computed as averages for the lowest 50 m AGL within each horizontal grid cell.

## 3. Results

### 3.1. Meteorological Fields

**Figure 2.**
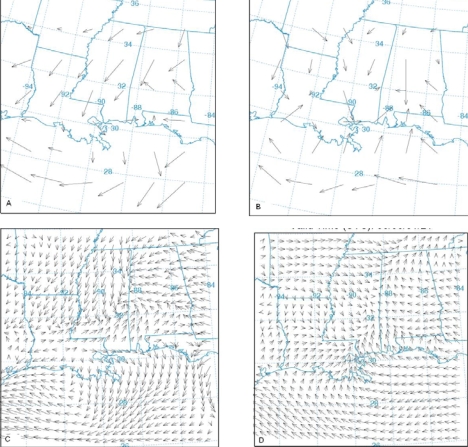

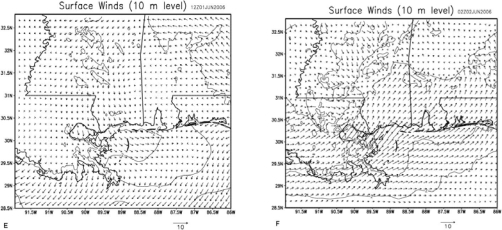
Surface horizontal wind field in Mississippi region on June 1, 2006 in the case of FNL analysis (a,b), Eta AWIP analysis (c,d), WRF model outputs (e,f) corresponding to 12 UTC (06 LT) 01 June 06 and 02 UTC (20 LT) 02 June 06 respectively. The arrow size in each case corresponds to a maximum wind of 10 m s^−1^.

Calculations from dispersion models are limited by the nature of the input meteorological data such as the resolution and representation of local terrain features in the low-level wind pattern [8]. The surface wind from the three input data sets are analysed first. Horizontal wind fields from the three meteorological data sets are shown in Figure 2 at 12 UTC (06 LT) and 02 UTC (20 LT) corresponding to the night and day time conditions. Surface wind in FNL data has coarse resolution. It is easterly over the sea region and northeasterly over the land region. Slight shift in wind flow to the land is seen in the daytime (Figures 2a,b). Wind field in the EDAS analysis seems to be represented better and more resolved indicating spatial variations than the FNL data. In a few grids near to the coast wind is northerly in the night and southerly in the day time indicating the diurnal variation in the flow pattern near the MS coast (Figures 2c,d). Simulated wind flow from WRF model (Figures 2e,f) is well resolved and more clearly represents the diurnal pattern of winds than the FNL and the EDAS data. It depicts the topographic variations in the flow expected from the presence of complex coast line of Louisiana.

**Figure 3.**
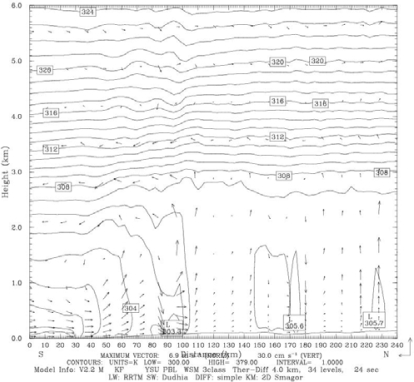
WRF simulated vertical cross-section of potential temperature and circulation vectors at 15 LT, 1 June 2006.

**Figure 4.**
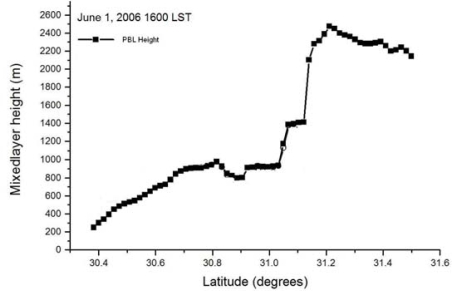
Simulated boundary layer height across the MS coast at 10 UTC (16 LT), June 01, 2006. The latitude of the coast is 30.4° N.

**Figure 5.**
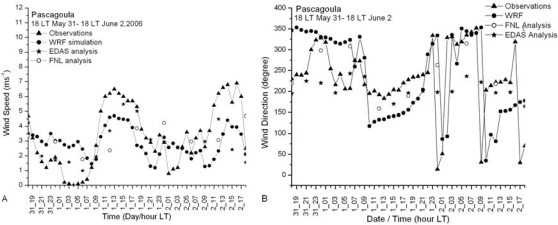

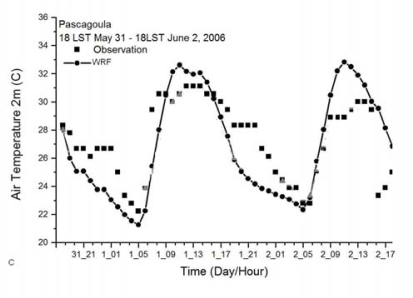
Diurnal pattern of observed and simulated surface (10 m) wind speed (A), wind direction (B) and 2m Air Temperature (C) at Pascagoula station from 00 UTC June 01 – 00 UTC June 02, 2006.

Results of the meso-scale model simulation are given in more detail in [17]. Here a few results from that simulation relevant to the dispersion study are presented. During the morning time the surface wind over Mississippi and Louisiana is northerly. It gradually becomes north-westerly offshore and gains in strength especially in parts of Mississippi and Louisiana coastal plains indicating prevalence of land breeze circulation. The surface wind at the coast turns southerly around 11:00 LT indicating development of sea breeze. Simulated sea breeze penetrates more inland in the subsequent times (Figures 2e,f). The maximum wind speed increases to about 5 ms^−1^ near the coast at 16:00 LT, the extent of the sea breeze was up to 50 km at this time. The flow is onshore and extended deep inland in the night. The simulated vertical cross-sections of wind and potential temperature at 15:00 LT at 89.75° E are shown in Figure 3. Circulation near the coast line at distance point (=100 km from south) shows development of a sea-breeze front with convergence, ascending winds at the leading edge, return flow aloft and subsidence behind the front. Horizontal circulation associated with sea breeze is seen to prevail up to a height of about 1,000 m. Temperature contours across the coast indicate stably stratified layers in the lower atmospheric region in the morning time on the sea and land regions, which gradually transforms to unstable stratification over the land in the afternoon time at 15:00 LT (Figure 3). A shallow boundary layer near the coast and deep boundary layer in the inland region are seen. The shallow mixing layer extends horizontally up to 30 to 40 km inland. Simulated PBL height across the coast also shows a shallow PBL near the coast and a deep boundary layer inland (Figure 4). Sea breeze extends up to 80 km inland and mixed layer depth across the coast varies from 200 m to 800 m. Diurnal variation in modeled and observed near surface (10 m) wind speed, wind direction and air temperature (at 2m) along with corresponding values from FNL and EDAS data for the period, June 01–03, 2006 are shown in Figure 5 at Pascagoula Mesonet tower located at 30.36 N and −88.52 E roughly at a distance of 5 km from the coast. Both observations and model values at Pascagoula indicate increase in wind speed, decrease in air temperature and shift in wind direction around the noon time indicating sea-breeze onset. A shift in wind direction from 275 (northwesterly) to 200 (southerly/ southwesterly) is found both in the model values and observations. The model could reproduce the observed trends of the surface variables (Figure 5). The data from EDAS and FNL follow the trend in observations to some extent. Although the EDAS and FNL data follow the order of the parameters, the diurnal cycle is better represented in the WRF simulated fields.

### 5.2 Forward Trajectories

**Figure 6.**
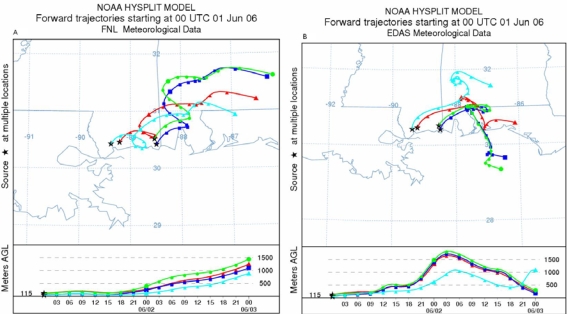

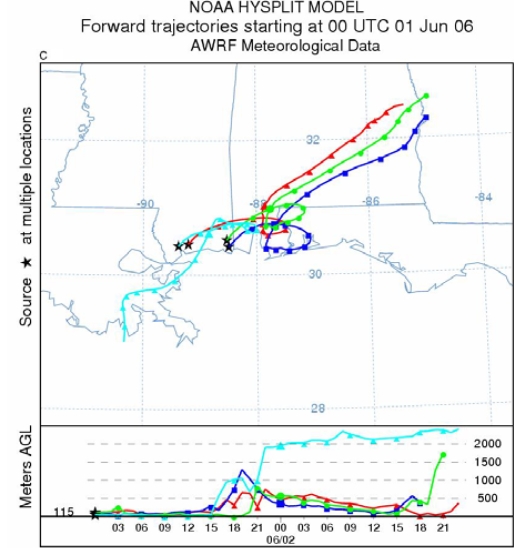
Forward trajectories from the source regions with FNL analysis (a), Eta AWIP analysis (b) and WRF model outputs (c).

Simulated forward trajectories from the release locations are noticed to vary with each case of meteorological data (Figure 6). Trajectories with FNL data move in the northeast direction and no significant back turning is found. Trajectories in the cases of EDAS and WRF data sets also move in north east direction but show looping to the coast indicating effect of onshore and off-shore flows on the movement of air parcels. The looping of the trajectories is clearer from the trajectories drawn with WRF wind fields. Trajectories in these two cases also show vertical growth while moving inland due to variations in the vertical mixing across the coast.

### 5.3. Plume Distribution Pattern

**Figure 7.**
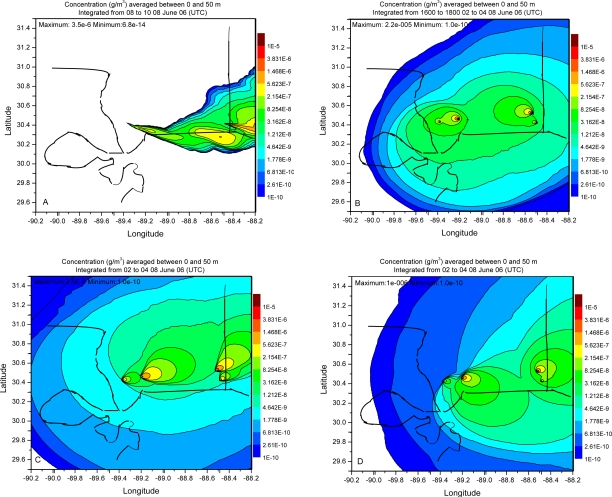
Simulated SO_2_ plume concentration distribution using FNL data at 08–10 UTC, 01 June 06, 16–18 UTC 01 June 06, 02–04 UTC 02 June 06 and 20–22 UTC 02 June 06 respectively.

**Figure 8.**
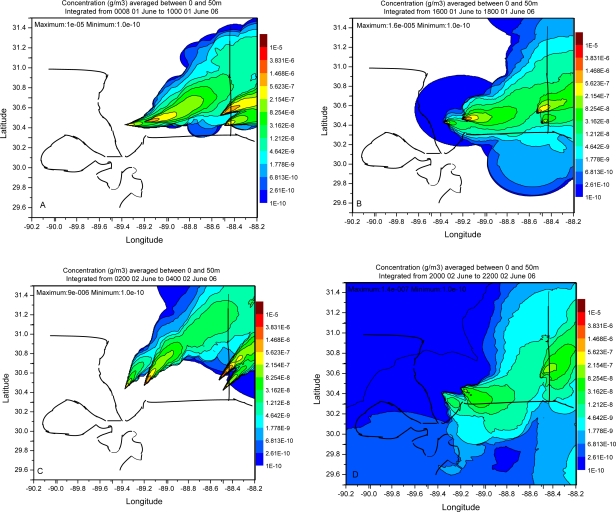
Simulated SO_2_ plume concentration distribution using Eta AWIP data at 08–10 UTC, 01 June 06, 16–18 UTC 01 June 06, 02–04 UTC 02 June 06 and 20–22 UTC 02 June 06 respectively.

**Figure 9.**
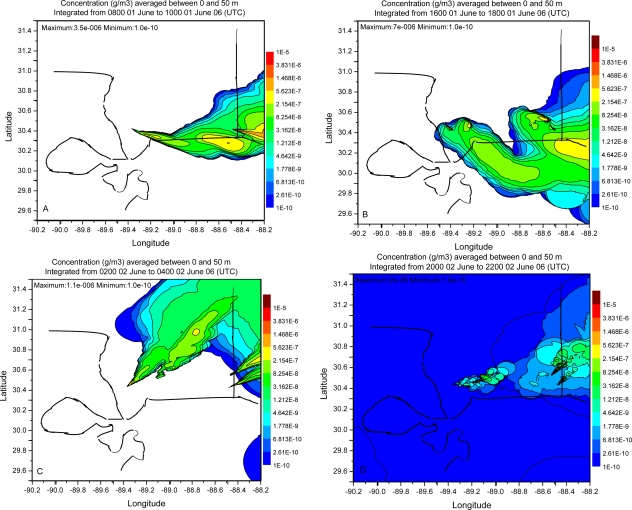
Simulated plume concentration distribution using WRF model data at 08–10 UTC, 01 June 06, 16–18 UTC 01 June 06, 02–04 UTC 02 June 06 and 20–22 UTC 02 June 06 respectively.

Dispersion simulation in the present work is evaluated following the procedures outlined in [7,8]. A visual examination of the plume distribution pattern is made followed by statistical analysis. Figures 7, 8 and 9 show contours of model calculated 2 h average ground level concentrations in the case of simulations with FNL, EDAS and WRF meteorological fields respectively. Each of these figures shows the concentration distributions for the periods ending at 10, 18, 28 and 42 h respectively after the beginning of calculation. All the plots contain the same concentration contour intervals. Visual comparison shows the spatial pattern of concentration field is distinctly different in the three cases of meteorological data. Calculations with FNL data show relatively uniform distribution of the plume around the sources and the individual plumes from the sources are difficult to be identified. Also diurnal alteration in the plume direction was not significant in this case. Orientation of the plume was mostly to the west and followed the large scale wind flow pattern in the case of simulation with FNL data. Simulations with EDAS meteorological fields showed diurnal alteration in the plume direction to some extent but was largely influenced by the synoptic flow.

Plume pattern in the case of simulation with WRF meteorological fields clearly showed the diurnal alternation in plume direction according to diurnally varying wind flow pattern. Wide spatial and temporal variability of the plume is noticed in the calculations with WRF fields. Also the individual plumes from each of the sources could be clearly seen in the downwind direction in the cases of WRF and EDAS fields. The plume direction was to the east at early morning time (Figure 9A), to the southeast in the noon (Figure 9B), to the north east (Figure 9C) in the evening / night conditions and to the east-northeast in the afternoon next day (Figure 9D) in the case of simulation with WRF fields. In the simulation with WRF fields, the concentration pattern during offshore wind condition shows the ranges (2.15e-7 – 8.25e-8), (1.48e-6 – 5.62e-7) extended to larger downwind distances over the oceanic region. Similarly the concentration pattern during the night condition (Figure 9C) showed the range (2.15e-7 – 8.25e-8) extended to large distance over land.

The concentration distribution shows that the concentration maximum occurred near the source in the cases with FNL and EDAS data fields whereas it occurred away from the source in the case of simulation with WRF. The iso-concentration contours 3.83e-6, 1.47e-6, 5.62e-7, 2.15e-7, 8.25e-8 are seen to extend to larger downwind distances in the simulation with WRF fields as compared to the simulation with EDAS data. Statistics of the concentration distribution in dispersion simulations of different meteorological data are given in table 4. The maximum, mean and standard deviation of the concentration for the four sampling times (08–10 UTC June 01, 16–18 UTC June 01, 02–04 UTC June 02, 20–22 UTC June 02) are less in the cases of simulation with EDAS and WRF meteorological fields than with FNL fields. The mean and range of concentration values are high in the simulation case with WRF fields. The relatively uniform concentration distribution in FNL plume prediction is perhaps due to the reason that the horizontal dispersion rate in the method followed depends on the spatial resolution of the meteorological data. This is related to the under sampling of the flow field as seen from the wind vectors (Figures 2a,b) which change dramatically between the grid points along the coast line due to unresolved smaller scale features. The relatively coarse grid FNL data suggests that the horizontal deformation values will be much larger than in the WRF or EDAS data which are not under-sampled and have a more consistent pattern in the wind vector change between grid points. Thus the high resolution WRF data has improved the horizontal dispersion over the relatively coarse FNL and EDAS meteorological data fields.

**Figure 10.**
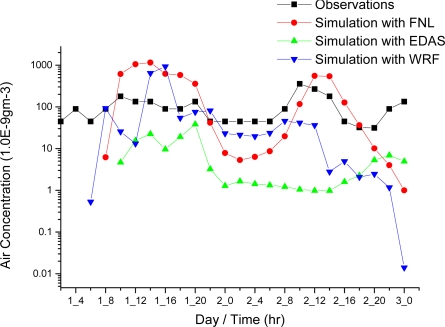
Diurnal variation of observed and simulated SO_2_ concentration at Pascagoula monitoring station.

Figure 10 shows observed and model concentrations sampled at Pascagoula monitoring station. It is to be noted that the SO_2_ observations include both the background and longer range contributions from other nearby sources as well which are not included in the model calculations. The model values show contributions only above the background. Hence the observations are generally higher than the model values. However the observations are used here merely to compare the trends in the simulated values. Model calculations in all the three cases show diurnal variation in concentration. Calculations in the initial 6 h period are below observations in all the three cases due to grid instauration.

Model concentrations with WRF data are closer to observations up to 40 h and deteriorate thereafter. Statistical parameters of correlation and fractional bias are calculated between observed and calculated concentrations. Correlation (R) is used to represent the scatter among paired measured and predicted values and a fractional bias (normalized bias) (FB) is used to indicate over-prediction or under-prediction [7]. Calculated concentrations in all cases show an increase in bias towards the tail of simulation. Simulated concentrations with Eta analysis are an order less than those with FNL and WRF fields. Calculations with FNL fields are overestimated during day time and underestimated during night time. This is seen in the positive fractional bias during 8 h daytime period and negative fractional bias during 8 h night time period (Table 5). An overall (24 h) high correlation and positive bias is noticed with FNL data. The higher correlation in FNL plume prediction is probably because of the closeness of the wind speed, direction and other meteorological parameters in FNL data with the observations over 6 h intervals and because the FNL values remain constant in the dispersion simulation over every 6 h period unlike the EDAS and WRF data. However the WRF plume provides a more realistic representation of the plume diurnal cycle.

**Figure 11.**
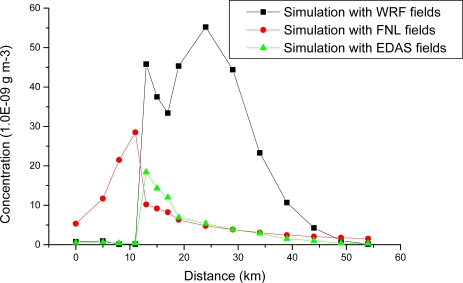
Simulated downwind concentration of SO_2_ with different meteorological fields at 02–04 UTC 02 June, 2006.

Higher concentrations observed in coastal regions are generally associated with sea-breeze circulation and shallow mixing during thermal internal boundary layer formation. Downwind concentrations in the three simulations for the afternoon time (18 LT) are shown in Figure 11 when the plume is spread completely over land. While the downwind concentration falls monotonously with FNL and Eta EDAS data some difference is noticed in the case with WRF fields. Maximum concentrations are noticed at distances of 10 – 40 km in the afternoon time coinciding with sea-breeze time and the consequent shallow mixing layer formation and during the night conditions. Figure 9C shows the concentration contours 5.6e-7, 2.15e-7, 8.25e-8 extend to large downwind distances in the north east direction during the night time in the dispersion simulation with WRF outputs unlike in the other two cases.

## 6. Conclusions

This study explores the impact of using three different meteorological inputs on atmospheric transport and dispersion model solutions calculated by the HYSPLIT model. A case study of 48-h simulation along the coastal region of the Gulf of Mexico is investigated. A chemically non-active tracer is released in HYSLIT from four sites in the coastal zone of MS, with mass corresponding to reported emissions of SO_2_. Simple statistical verification is done against available SO_2_ measurements in the study domain. An attempt is made to correlate differences in input wind fields with diurnal trends of observed and HYSPLIT SO_2_ concentrations.

Simulations with the three data sets clearly indicated differences in the estimated trajectory, plume movement and the concentration patterns. Among the three cases, the predictions from WRF model reveal presence of meso-scale land/sea breeze circulation and associated mixed layer alterations in the Mississippi Gulf coast. The Eta EDAS analysis could show these characteristics to some extent where as they are not represented in the FNL analysis data. Plume pattern with NCEP FNL analysis data followed the large scale flow. The effects of meso-scale sea breeze and IBL effects are not found in the case of calculation with FNL data and EDAS fields. The flow fields from WRF could better simulate the diurnal plume distribution pattern than the other two data sets, which may be due to the resolving ability of the mesoscale model while using a 4-km grid over the study area. Calculations with WRF data clearly show plume recirculation due to sea breeze and follow observed trends of concentration. The relatively uniform plume distribution in the case of FNL is probably due to the large horizontal deformation associated with the coarse wind field data in the case of FNL analysis. The plume distribution in the coastal area is better resolved in the cases of EDAS and WRF fields which could more realistically represent the local scale flow field. There were some deviations in the simulated concentrations in the case of WRF fields in the initial 6 hours which may be because of the model adjustment and spin up to the topography. The deviations in the concentrations are an order more during sea breeze time than during other times (10^−6^ to 10^−7^ gms^−1^). While the downwind concentration falls monotonously in the simulations with FNL and Eta EDAS data, concentrations are noticed to reach maximum values at 25–40 km distance range during the night time and in the afternoon times in the simulations using WRF fields. This may be because of the formation of shallow mixing region during the afternoon and night hours in the WRF simulation. The current study shows improvement in local scale dispersion simulation with the meso-scale analysis data (Eta EDAS) and more specifically using meso-scale fields from WRF fields, especially in plume distribution in the coastal environment. The species used is actually a reactive chemical species (SO_2_) with contributions from many local and distant sources which makes the model evaluation subject to too many unknowns. However, considering the unknowns common in the three cases, the verification in the present study only focused on general trends. Also the present study uses observations from a single monitoring site for verification. However to distinguish between the influences of the meteorology versus those of subgrid processes in HYSPLIT,more number of verification sites need to be considered, which will be attempted in the future studies. The current study shows in a general way the problems associated with the use of analyses (FNL and EDAS) to represent evolving meteorology over a 48 h period for the atmospheric transport and dispersion applications, especially the inability to represent synoptic-scale and mesoscale vertical motion fields (viz., sea breeze) in a way that is consistent with the horizontal flow field. Also more cases would be required to determine the relative skill when using the three cases of input meteorological data sets.

## Figures and Tables

**Table 1.** Details of the physics and grid configuration used in WRF model.

**Dynamics** **Vertical resolution**	Primitive equation, non-hydrostatic 35 levels		

**Domains**	**Domain1**	**Domain2**	**Domain3**

**Horizontal resolution**	36 km	12 km	4 km
**Grid points**	54 × 40	109 × 76	187 × 118
**Domains of integration**	93.0 W – 78.05 E 27.16 N – 34.45 N	91.74 W – 81.92 W 28.5 N – 34.45 N	90.28 W – 84.77 W 29.38 N – 32.54 N

**Radiation**	Dudhia (1989) scheme for short wave radiation, Rapid radiative transfer model (RRTM) for long wave radiation		
**Surface processes**	5 layer soil diffusion scheme		
**Boundary layer**	Yonsei State University (YSU) PBL scheme		
**Sea surface temperature**	NCEP FNL analysis data		
**Convection**	Kain and Fritsch scheme on the outer grids domain1, domain2		
**Explicit moisture**	WSM3 class simple ice (SI) scheme		

**Table 2.** Sources of elevated release considered for the HYSPLIT computations.

Elevated Source Location	Source	Latitude / Longitude	Stack Height Hs (m)	Stack Diameter Ds (m)	Source strength Qs (g s^−1^)

Gulfport (A)	Mississippi Power Plant Jack Wa	30.46° N; −89.21° E	115.12	3.85	24,869.5
Pascagoula (B)	Chevron Products Pascagoula Refinery	30.42° N: −88.49° E	54.1	1.35	1,742.8
Escatawpa (C)	Mississippi Power Plant Victor	30.52° N; −88.53° E	105.0	10.23	12,522.2
Passchritian (D)	Dupont Delisle Facility	30.43° N; −89.38° E	45.0	3.0	1,270.5

**Table 3.** HYSPLIT dispersion model configuration.

**Model version**	4.8
**Grid Centre**	30.5 N, −89.5 L
**Vertical resolution**	8 Levels – 25, 50, 100, 200, 500, 1000, 2000, 5000
**Horizontal Grid**	2 × 2 degree
**Horizontal resolution**	0.01 × 0.01
**Turbulence Method**	Standard Velocity Deformation
**Meteorology**	NCEP FNL 6 h data, EDAS 3 h data, WRF Simulated hourly meteorological fields
**Frequency of emissions cycle**	500 particles per hour

**Table 4.** Statistics of the dispersion calculation with different input meteorological data. (S.D- standard deviation, S.E- Standard error), units are in g/m^3^.

Data Used	Time (UTC)	Statistics of dispersion calculation (units : g/m^3^)					
		Mean	S.D	S.E	Min	Max	Range

FNL	08–10 June 01	1.01E-08	5.43E-08	1.35E-10	0.0	3.28E-06	3.28E-06
	16–18 June 01	8.90E-09	8.91E-08	2.22E-10	0.0	2.05E-05	2.05E-05
	02–04 June 02	1.27E-08	5.96E-08	1.49E-10	0.0	3.32E-06	3.32E-06
	20–22 June 02	8.53E-09	6.19E-08	1.54E-10	0.0	1.15E-05	1.15E-05

ETA AWIP	08–10 June 01	8.73E-09	5.81E-08	1.45E-10	0.0	9.70E-06	9.70E-06
	16–18 June 01	5.69E-09	6.64E-08	1.66E-10	0.0	1.45E-05	1.45E-05
	02–04 June 02	6.42E-09	6.74E-08	1.68E-10	0.0	8.17E-06	8.17E-06
	20–22 June 02	3.11E-09	9.12E-09	2.27E-11	0.0	1.24E-07	1.24E-07

WRF	08–10June 01	1.01E-08	5.43E-08	1.35E-10	0.0	3.28E-06	3.28E-06
	16–18 June 01	1.43E-08	5.72E-08	1.43E-10	0.0	6.93E-06	6.93E-06
	02–04 June 02	7.28E-09	2.53E-08	6.32E-11	0.0	1.01E-06	1.01E-06
	20–22 June 02	5.47E-10	1.01E-08	2.51E-11	0.0	1.95E-06	1.95E-06

**Table 5.** Results of dispersion calculations with FNL, EDAS and WRF meteorological data. R- correlation coefficient, FB- fractional bias.

Meteorological data	R	FB (24 h)	FB (8 h day)	FB (8 h night)

FNL	0.396	0.81	1.47	−1.04
EDAS	0.10	−1.33	−1.39	−1.85
WRF mesoscale simulation	0.21	−0.82	−1.47	−0.41
